# Effectiveness of the online-eLearning program KeepCoool at improving the vaccine cold chain in general practices

**DOI:** 10.1371/journal.pone.0301847

**Published:** 2024-04-16

**Authors:** Anika Thielmann, Marie-Therese Schmitz, Thomas Welchowski, Birgitta Weltermann

**Affiliations:** 1 Institute for Family Medicine and General Practice, University Hospital Bonn, Bonn, Germany; 2 Department of Medical Biometry, Informatics and Epidemiology, Faculty of Medicine, University of Bonn, Bonn, Germany; Ministry of Health, Sri Lanka, SRI LANKA

## Abstract

**Background:**

Protecting vaccines from freeze damage is a poorly addressed problem. We describe the effectiveness of the eLearning KeepCoool on cold chain maintenance in general practices.

**Methods:**

For this intervention study, temperatures of vaccine refrigerators were logged at one-minute intervals. Personnel from practices with cold chain breaches was offered the eLearning. The primary outcome was the intervention’s effectiveness to achieve temperatures in the target range (2 to 8°C) in the sixth week (follow-up) compared to the first (baseline). Using continuous temperature data, a generalized additive model for location, scale and shape was estimated.

**Results:**

The practice response rate was 38% (64 of 168). At baseline, 73% of the practices and 68% of the refrigerators (51 of 75) showed cold chain breaches. 47% of the practices (n = 22 with 24 refrigerators) participated in the eLearning (55 physicians and practice assistants). At follow-up, 17% of those refrigerators were in the target range continuously, 38% reached targets 95% of the time while always >0°C, and temperatures ≤0°C decreased by 63%. Based on 2 million temperature data, the average Euclidian distance based on regression showed a significant improvement (p<0.05).

**Conclusion:**

The eLearning KeepCoool improved the practices´ vaccine cold chain. It is freely available at https://keepcoool.ukbonn.de.

## Introduction

The World Health Organization (WHO) considers protecting vaccines from freeze damage ‘one of the most poorly addressed problems in vaccine management’ [1]. Cold chain breaches are documented worldwide [2] and linked to disease outbreaks [3–5] or suspected to be linked [6]. In moderate climates, freeze exposure is the key problem which is especially dangerous to adsorbed vaccines (e.g. hepatitis B, tetanus, diphtheria, pertussis): aluminum-containing adsorbents form irreversible precipitates which decrease vaccines’ potency and may induce local irritation [7, 8]. For example, absorbed hepatitis B vaccines have a freezing threshold of -0.5°C [8]. To ensure vaccines potency, most vaccines are to be maintained within a temperature cold chain between +2°C and +8°C starting from the time of manufacture till preparation for patient application. Recently, new mRNA vaccines have increased the attention for the cold chain as some require -70°C for long-term stability and are allowed for storage at +2°C to +8°C for some weeks only [9].

Deficits in vaccine refrigerator management are documented worldwide. A systematic literature review showed freezing temperature exposure in 33.3% of refrigerators used for vaccine storage in ten wealthier countries [2]. Additional deficits were the lack of thermometers (6.9% to 91.9% of refrigerators) [10–17] and temperature logbooks (26% to 94%) [11, 14, 16]. Also, 20.3% to 52.6% of the practices incorrectly stored vaccines in door shelves [12, 13, 17]. In a 2014 survey among German general practitioners (GP), 16% reported cold chain breaches as error or near error, and only 51% reported to document refrigerator temperatures twice a day as recommended [18, 19].

Aiming at improving cold chain maintenance in general practices, we developed and evaluated the guideline-based eLearning program KeepCoool for GPs and practice assistants. The elearning was based on recommendations from several countries (US [20], UK [21], Australia [22], Canada [23], Scotland [24]). It provides learning content in five tutorials: temperature, refrigerator, storage, responsibilities, and monitoring. When starting, participants completed an 11-item knowledge questionnaire. The effectiveness of the elearning was evaluated in a prospective study with 64 general practices addressing knowledge and refrigerator temperatures. As published, the prevalence of refrigerators with temperatures in the target range was 32% at baseline [25]. Also, a high prevalence of additional storage deficits was documented, e.g., no use of bins (81.3%), no thermometers in the center of refrigerators (54.0%), vaccines boxes with contact to outer walls (46.3%), refrigerator unsuitable for vaccine storage (44.6%), and unwrapping of vaccines (31.1%) [26]. Access to the eLearning program KeepCoool significantly increased the knowledge of GPs and practice assistants: the mean number of correct answers was 5.6 of 11 answers at baseline and increased to 9.8 after program participation (p<0.001) [27].

Drawing on further data of the KeepCoool study, we here describe the effectiveness of the eLearning program KeepCoool to improve the vaccine cold chain measured by continuous temperature logging after six weeks. Secondary outcomes comprised chances in the prevalence of freezing temperatures, refrigerators used, and a Euclidian prediction model using continuous temperature data.

## Methods

### Study population and study design

The KeepCoool study was conducted in German general practices from a university teaching practice network (n = 185) from February to October 2018. Typically, these physician-owned practices are one to three physician practices with about 2 practice assistants per physician with larger group practices currently emerging. The practices serve patients covered by the statutory health insurance (about 90% of the population) and private insurances (about 10% of the population). Practices frequently have long lasting relationships with their patients.

Recruitment followed a structured approach: contact by phone and fax up to three times or until practices responded. A trained health services researcher (A.T.) and a trained study assistant visited all practices which had volunteered for participation. They had been involved in other practice studies and were familiar with the specifics of the setting as well as the topic. They informed the practice teams about the study and the study materials, obtained written informed consent, conducted the baseline survey, and placed the data logger in the refrigerators. Details are published [25, 26, 28].

The study was originally designed as a randomized, controlled trial with intervention arm (study arm 1) and waiting-list control arm (study arm 2) in practices with confirmed cold chain breaches [28]. In addition, the original design included a third arm to follow practices with temperatures in the target range (study arm 3: monitoring only). However, temperature measurements of the first 7 days (baseline) were far worse than expected: 68% refrigerators were outside the target range, and 15% had reached critically low temperatures <0°C for up to 7 days [25]. Subsequent ethical considerations motivated the change to a study with pre-post-design to avoid the use of vaccines with reduced potency and to assure patient safety. Thus, the study arms 1 and 2 were combined resulting in a total of two study arms: intervention arm (A) with practices having cold chain breaches at baseline. The monitoring only arm (B) with practices without cold chain breaches at baseline remained identical (former study arm 3). Thus, access to the eLearning program KeepCoool was offered to all practices with cold chain breaches at baseline (intervention arm A). For analyses, the practices in the intervention arm A were later grouped according to their participation or non-participation in the eLearning. We aimed to investigate if the participation in the eLearning improved refrigerator temperatures. Data logging remained identical for all practices. Temperature data logging was continued every minute until the follow-up practice visits after a minimum of five weeks.

For analysis, refrigerators served as their own control. Separately, refrigerators in the target range at baseline were monitored to evaluate if temperatures were maintained (monitoring only). The responsible ethics´ committee was informed on the protocol change. All practice sites originally included remained in the study.

### eLearning intervention KeepCoool

The best practices-based eLearning KeepCoool provides learning content in five tutorials: temperature, refrigerator, storage, responsibilities, and monitoring. When starting, participants completed an 11-item knowledge questionnaire as basis for tailored learning. The eLearning used three didactic elements: a) personal address, b) targeting of the respective profession (physicians or practice assistants), c) individualized feedback tailored to a participant’s baseline knowledge. The program followed a consistent presentation with `basic information´, `tips for the practice´, and `expert knowledge´ for in-depth understanding. Options to download relevant literature and practice templates were available. After the eLearning, participants who successfully answered ≥ 7 of the same 11 questions (> 60% correct answers as required by the regional physician association) received a certificate. For details [27].

### Continuous temperature logging, primary and secondary outcomes

Temperatures were measured using a data logger (testo 175T) with an accuracy of ±0.4°C in the operating range -5°C to +10°C (calibrated under a DIN EN ISO 9001:2008 certified quality assurance system). The device was equipped with a standard probe which measured ambient air temperatures inside the refrigerator at 1-minute intervals similar to prior studies [10, 12, 15, 29, 30]. We abstained from glycol probes [28] due to limited resources and an easier handling of the new device, which remained inside the refrigerators throughout the study with the display turned off and memory access locked [25]. For the analyses, the first 120 minutes after each data logger set-up were excluded to allow for probe acclimatisation.

Using data from all practices, the effectiveness of the intervention was determined using 7-day-temperature readings (10,080 recordings) of the 1^st^ week (baseline period) compared to the 6^th^ week (follow-up period). The primary outcome was the percentage of refrigerators with temperatures within the target range (2 to 8°C) in the follow-up period. Secondary outcomes comprised various parameters, e.g., the prevalence of freezing temperatures (see Table 2), structural changes of refrigerators, and a Euclidian temperature prediction model. For the three temperature zones (+2 to +8C; ≤0°C; ≤ -0.5°C) we calculated the hours in the respective zone (so called: cumulative time) and the longest period in the respective zone (so called: longest consecutive time). In addition, we noted if—and when—the practice assistants and the physicians finished the KeepCoool eLearning knowledge test and received the certificate.

### Ethical approval, consent to participate and trial registration

The study complies with the ethical principles of the World Medical Association Declaration of Helsinki. Ethical approval was obtained from the Ethic Commission of the Medical Faculty of the University of Duisburg-Essen (reference number: 14-6118-BO). Participants provided written informed consent. The study was registered in the German Trial Register: DRKS00006561 (date of registration: 20 February 2015).

#### Statistical analyses

A sensitivity power simulation was conducted with 1055 observations randomly drawn with replacement of the complete data set. Then the temperatures of the refrigerators were randomly drawn from the response distribution conditional on the estimated parameters from the original model M3. After data generation, model M1 was estimated with the same parameter specification as model M3. In addition, model M2 had the same specification, but without intervention main and interaction effects (null hypothesis). Those two nested models M2, M3 were compared with a likelihood-ratio test. The complete procedure was repeated independently 250 times and the intervention effect was detected in all simulated cases. This shows that the much larger total sample size of 2110282 is more than enough to achieve high levels of power to detect the intervention effect.

Practice, refrigerator, personnel, and temperature characteristics were analysed using descriptive statistics comparing the first week (baseline) with the sixth week (follow-up). In addition, all available temperature measurements observed (up to 20 weeks) were used for two analyses:

1. To account for participants´ variations in accessing the eLearning, the periods before/after the first participant finished the test were compared regarding the percentage of time within the target range (2 to 8°C).
2. Due to the high temperature variability between refrigerators and a target range of 2 to 8°C centred around 5°C, we chose a generalized additive model for location, shape and scale [31] to identify if a practice’s participation in the eLearning changed/improved temperature over time. This approach has the benefit to not only model a mean response, but also the variance depending on the co-variates. The trend over time was estimated with a p-spline basis [32]. Besides the main intervention effect all second and third order interactions between intervention and the other fixed effect variables (e.g., temperatures measured on weekends, physician participation, number of participants per practice, group versus solo practice) were included. Heterogeneity between practices and refrigerators were taken into account as random intercept effects. Due to the large sample size model selection of the distribution of the temperature was conducted without covariate effects based on the generalized Akaike information criterion [33]. As result the skewed power exponential type four distribution was chosen [34, 35]. The calculated average Euclidian distance compares the distance of the prediction interval to 5°C as median of the target range (2 to 8°C) for the time periods before and after the first participant per practice finished the test. The +5°C value was chosen because guidelines recommend to target 5°C when adjusting refrigerators. The t-test statistics was applied (p<0.05). The details are presented in the Supplement.

Statistical analyses were performed using IBM SPSS Statistics for Windows, version 25 (Armonk, NY: IBM Corp.) and R, version 4.0.4. Percentages and mean values are reported for valid cases. Models were estimated using R package gamlss (Version 5.3–2).

## Results

### Characteristics of participating practices and practice personnel

**Fig 1:**
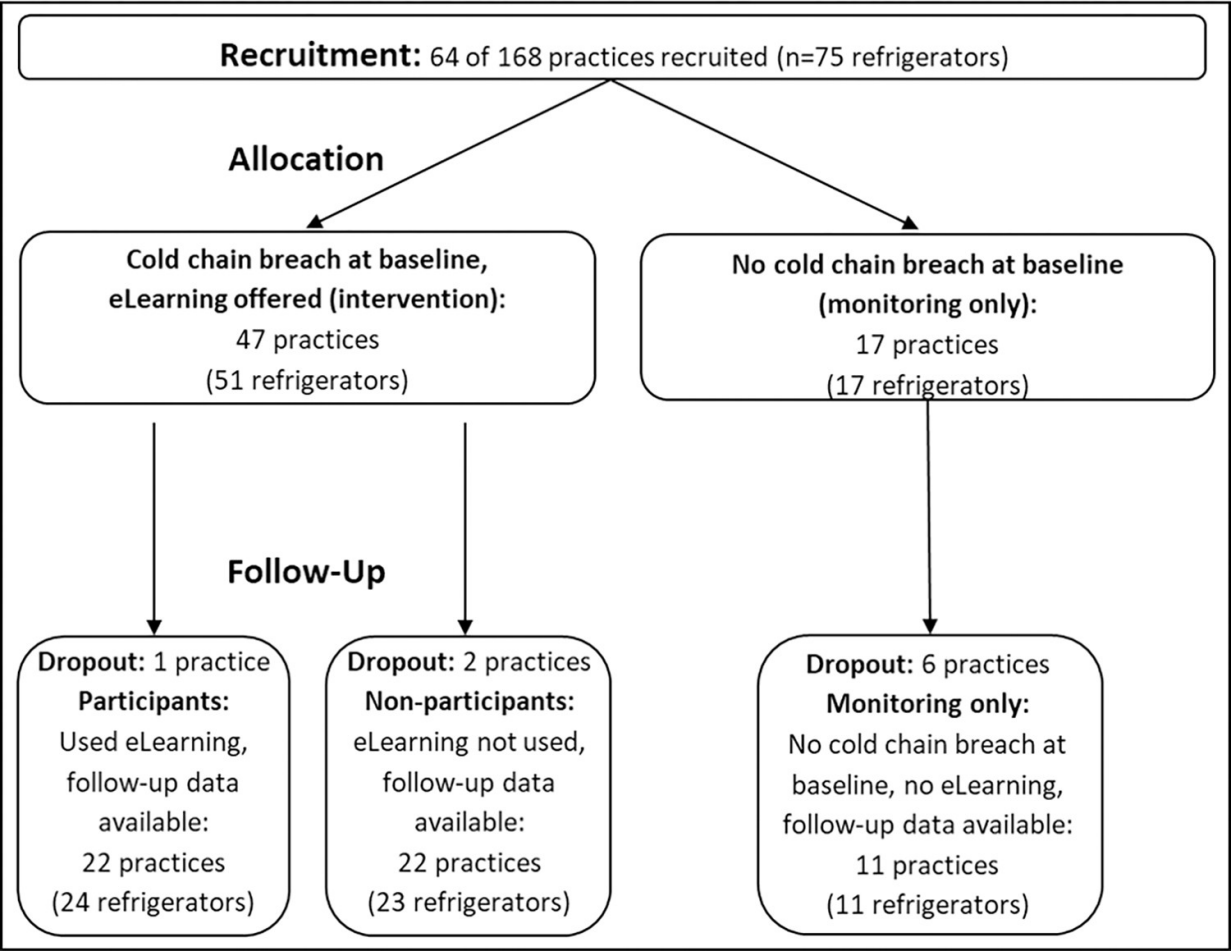
Consort flow-chart with overview on recruitment and follow-up (practices and refrigerators): Stratification by cold chain-breaches and eLearning participation (participants vs. nonparticipants) and monitoring only practices; practice drop-outs: 1 in participants, 2 in nonparticipants, 6 in monitoring.

Of the 168 practices contacted, 64 participated (response rate: 38.1%) with 75 refrigerators. At baseline, 73.4% of the practices (47 of 64) showed cold chain breaches in ≥ 1 refrigerator and were offered the eLearning. This offer was accepted by 48.9% of the practices (n = 23 of 47) (`participating practices´), while 24 practices did not participate (`non-participating practices´). Follow-up data were available for 44 of 47 practices (one drop-out among participating practices, two among non-participating ones). As separate third group, practices without cold chain breaches at baseline were followed (monitoring only practices). The subpopulations were comparable for key characteristics (Table 1 and Fig 1).

**Table 1:** Characteristics of practices and refrigerators stratified by practice subgroups.

	Total practices	Participating practices	Non-participating practices	Monitoring only practices
	(N = 55)	(N = 22)	(N = 22)	(N = 11)
		n (%)	n (%)	n (%)
**Practice characteristics**				
Group practice	30 (54.5)	11 (50)	15 (68.2)	4 (36.4)
Number of physicians in practice, mean [8]	1.9 (1.1)	1.9 ± 1.37	2.1 ± 0.94	1.6 ± 1.06
Number of practice assistants, mean SD [9]	4.7 (2.7)	4.1 ± 2.65	5.1 ± 2.73	5.0 ± 3.07
> 1,750 patients per practice per quarter (caseload) [9]	19 (42.2)	9 (50)	8 (40)	2 (28.6)
> 85% of patients with statutory health insurance [7]	31 (66.0)	15 (78.9)	10 (52.6)	6 (66.7)
Tropical medicine and/or yellow fever service [7]	4 (8.5)	1 (5.6)	2 (9.5)	1 (12.5)
**Refrigerator characteristics**				
Number of refrigerators	58	24	23	11
Types of refrigerators:				
• Pharmaceutical grade	4 (6.9)	0 (0)	2 (8.7)	2 (18.2)
• Household model	54 (93.1)	24 (100)	21 (91.1)	9 (81.8)
○ Freezerless refrigerator	22 (37.9)	11 (45.8)	8 (34.8)	3 (27.3)
○ Refrigerator with internal ice compartment (one exterior door)	27 (46.6)	12 (50.0)	10 (43.5)	5 (45.5)
○ Refrigerator with internal non-insulated ice compartment (one exterior door)	2 (3.4)	1 (4.2)	0 (0)	1 (9.1)
○ Full-size dual-zone refrigerator/freezer (separate exterior doors)	2 (3.4)	0 (0)	2 (8.7)	0 (0)
○ Household model without further details	1 (1.7)	0 (0)	1 (4.3)	0 (0)

Missing data in []

### Refrigerators and temperatures at follow-up

Overall, we observed improvements in refrigerators and temperatures, most of which were significantly better in the participants than the non-participants, although these practices did not reach the quality of the monitoring only group (Table 2).

**Table 2:** Refrigerator temperatures at baseline and follow-up: All practices (total) and stratified by practice subgroups.

	Total practices (N = 58 refrigerators)		Intervention group: Participating practices (N = 24 refrigerators)		Intervention group: Non-participating practices (N = 23 refrigerators)		Monitoring only group (N = 11)	
	Baseline	Follow-up	Baseline	Follow-up	Baseline	Follow-up	Baseline	Follow-up
**Temperature,** Mean, ±SD	5.3±3.1	5.7±3.2	4.5±3.8	6.1±3.2	6.3±2.4	5.5±4.5	5.2±1.0	5.5±1.8
**Min–max**	-4.0 to 12.2	-12.8 to 14.9	-4.0 to 12.2	-1.4 to 13.7	-2.4 to 12.1	-12.8 to 14.9	2.0 to 7.5	-2.2 to 10.6
**2**–**8°C**, N (%)								
• Always within target range: N (%)	11 (19.0)	15 (25.9)	0 (0.0)	4 (16.7)	0 (0.0)	3 (13.0)	11 (100.0)	8 (72.7)
• Cumulative time (in hours): mean±SD	117.2±52.7	112.0±65.0	88.0±55.7	101.2±71.6	123.2±41.3	104.0±64.9	168.0	152.4±29.3
• Longest consecutive time (in hours): mean±SD	67.5±67.6	69.9±70.6	36.5±48.1	66.9±67.9	51.9±55.5	47.8±60.3	168±0	122.6±75.0
**≤0°C (critically low at least once),** N (%)	10 (17.2)	8 (13.8)	8 (33.3)	3 (12.5)	2 (8.7)	4 (17.4)	0 (0)	1 (9.1)
• Cumulative time (in hours): mean±SD	70.8±58.0	72.4±74.4	83.2±57.7	108.5±92.3	21.2±28.0	55.1±70.9	0	33.5
• Longest consecutive time (in hours): mean±SD	42.6±54.4	56.7±72.6	51.6±57.8	97.1±86.4	6.6±7.4	40.1±65.6	0	1.8
**≤-0.5°C (critically low at least once),** N (%)	10 (17.2)	7 (12.1)	8 (33.3)	3 (12.5)	2 (8.7)	3 (13.0)	0 (0)	1 (9.1)
• Cumulative time (in hours): mean±SD	63.2±57.4	70.7±75.6	75.1±57.9	98.1±84.4	15.3±20.2	63.2±83.8	0	11.2
• Longest consecutive time (in hours): mean±SD	39.7±54.6	53.5±64.3	49.2±57.5	75.8±65.6	1.6±0.8	48.5±77.2	0	1.3
**Temperatures within the target range in 95% of the time and always >0°C,** N (%)	21 (36.2)	25 (43.1)	4 (16.7)	9 (37.5)	6 (26.0)	8 (34.8)	11 (100)	8 (72.7)
**Temperature results grouped in six exclusive groups,** N (%)								
1. Within target range but >8°C at least once	24 (43.1)	25 (43.1)	9 (37.5)	14 (58.3)	16 (69.6)	9 (39.1)	0 (0.0)	2 (18.2)
2. Always within target range 2–8°C	11 (19.0)	15 (25.9)	0 (0.0)	4 (16.7)	0 (100.0)	3 (13.0)	11 (100.0)	8 (72.7)
3. Within target range but at least once <2°C	16 (27.6)	7 (12.1)	11 (45.8)	0 (0.0)	5 (21.7)	7 (30.4)	0 (0.0)	0 (0)
4. Always >8°C	2 (3.5)	6 (10.3)	1 (4.2)	2 (8.3)	1 (4.4)	4 (17.4)	0 (0.0)	0 (0)
5. <2°, within target range, >8°C	3 (5.2)	3 (5.2)	2 (8.3)	2 (8.3)	1 (4.4)	0 (0.0)	0 (0.0)	1 (9.1)
6. Always <2°C	1 (1.7)	2 (3.5)	1 (4.2)	2 (8.3)	0 (0.0)	0 (0.0)	0 (0.0)	0 (0.0)

The primary outcome (continuous temperatures within target range of 2 to 8°C at follow-up) was reached by 4 of 24 (+16.7%) refrigerators. Improvement was noted also for secondary outcomes: critically low temperatures (≤0°C) 62.5% less at follow-up; in the target range in 95% of the time while always >0°C improved by 20.8% (baseline: 16.7%; follow-up: 37.5%).

Among participants, one practice had a new refrigerator (unsuitable at baseline) and one had ordered two refrigerators (both unsuitable at baseline). In the non-participants, one refrigerator was defrosted, one practice exchanged their refrigerator with another existing one, one bought a new refrigerator (unsuitable at baseline).

### Participation in eLearning and temperature changes

As published prior, the average knowledge score at baseline was 5.6 (SD ±1.9) of 11 correct answers with slight differences between physicians (5.9; SD ±2.3) and practice assistants (5.5; SD ±1.7) [27]. Average knowledge improved to 9.8 ± 1.2 with an increase of 4.2 points (p<0.001). In physicians, the mean knowledge score at follow-up increased by 4.6 points in physicians (p<0.001) and by 4.0 points in practice assistants (p < 0.001) [27].

**Fig 2:**
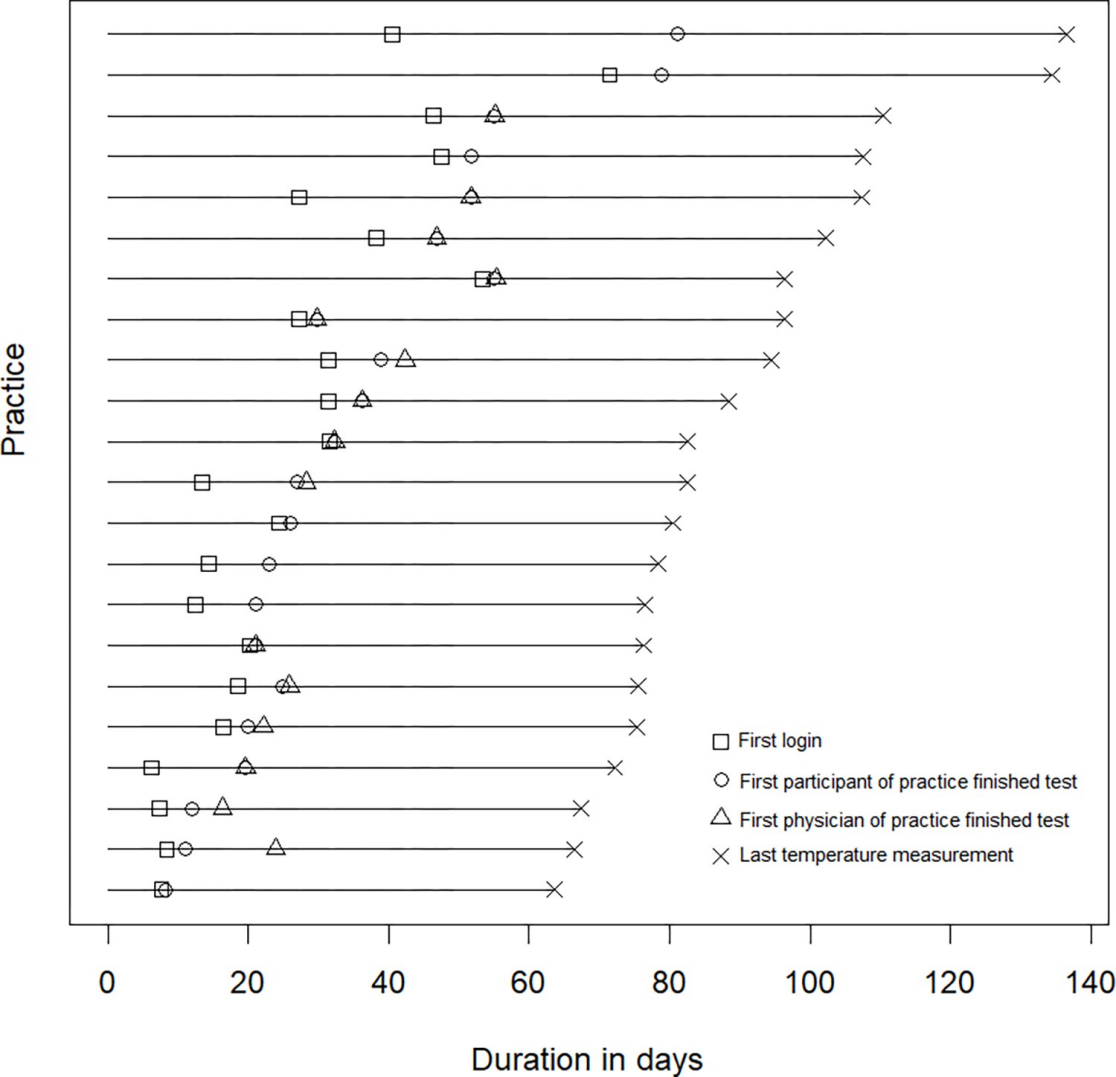
Timepoint of participation in eLearning stratified by practices (N = 22) and profession for the total temperature monitoring period (>3 months).

On average, the first person per practice finished the test 7.4 days (SD 9.16, range 0 to 40, median 5) after receiving the login information. Within practices the duration between the first and last participant who finished the test was 7.9 days (SD 11.93, range: 0 to 40, median 2). Seven practices finished eLearning during the sixth week follow-up or later. For details see Fig 2.

### Prediction model

**Fig 3:**
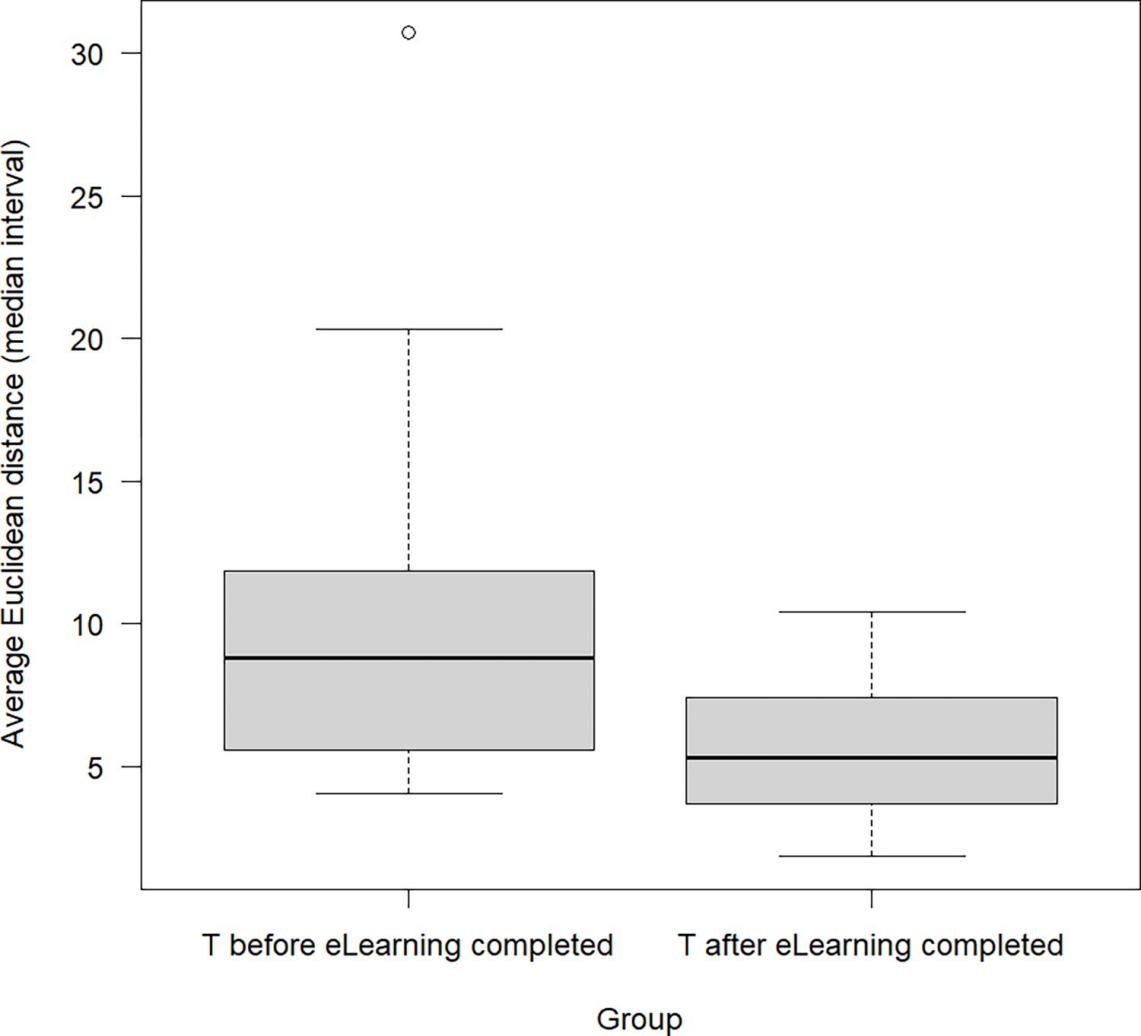
Refrigerator temperatures before and after eLearning: Average Euclidean distance of the 95% prediction intervals.

Fig 3 shows the average Euclidean distance of the 95% prediction intervals (considering both, model uncertainty and response uncertainty) from the two points (5, 5) up to and after the first participant finished the eLearning for the participating practices of the intervention group. These two points represent the average value of the interval between 2 and 8 degrees centigrade that is considered a good range for storage of medical vaccines. The smaller the Euclidean distances, the better are the circumstances for medical storage. Euclidean distances were significantly reduced after the first participant finished, as indicated by a paired t-test (p-value = 0.0046).

The model shows if a practice’s participation in the eLearning changed/improved temperature over time given the covariates observed time, weekend status, participation of a physician, number of participating practice assistants and single/group practice. Refrigerators of participating practices in the intervention group were analyzed using N = 2,110,282 single measurements (mean per practice 95,921.91, SD 22,445.52). All p-values were significant after multiple comparison adjustment regarding the false discovery rate [36] level of 0.05 except that weekend measurement did not influence the scale of the distribution. The intervention therefore affected both location and scale of the refrigerator temperature.

## Discussion

To our knowledge, KeepCoool is the first eLearning with proven effectiveness on vaccine storage knowledge [27] and cold chain management in the German speaking countries. Using the strict outcome of continuous cold chain maintenance in the 7-day follow-up period (2 to 8°C), 17% of refrigerators in the participating practices newly reached this target. Using more than two million temperature measurements over up to 4,5 months, our prediction model showed a significant improvement after participation in the eLearning KeepCoool.

In the international literature, few interventions with established effectiveness for improving the cold chain quality are available, none of which were based on eLearning. These interventions used various components either alone or combined: 1) written educational material, 2) introduction of thermometers with or without feedback on temperatures by either graphic display or telephone advice, 3) 1:1 onsite education with inspection of refrigerators. Direct comparison with KeepCoool is difficult due to methodological differences. With regard to reaching the target temperature range, the best result was observed in an Australian RCT of 50 primary care practices not yet using acceptable thermometers: onsite education with distribution of min/max-thermometers led to a fourfold increase of practices with refrigerator temperatures in the target range after 30 days [37]. Two other studies available provided long-term follow-ups after 6 months, 1 year, and 5 years: they documented a tendency for improved temperatures but also reported fluctuations over time in 25% of the practices with initially optimal temperatures [15, 29]. Similar fluctuations were observed in our monitoring only group (27.3%). In both foreign studies, the intervention comprised the distribution of national guidelines and serial feedback sessions with temperature graph and advice. In contrast to these interventions, our eLearning KeepCoool is much more cost-effective, yet additional reinforcement strategies are needed as practices with cold chain breaches improved significantly but only 13% of the practices were in the target range at follow-up compared to 72.7% in the monitoring only group.

In didactics, the one-size-fits-all approach is changing towards learner adaptive approaches [38]. One approach is tailored learning, which involves the individualization of information for each participants to reach better results regarding the desired behavior change. It is discussed that individualization increases the acceptability of information whereby information is more frequently remembered and discussed [39, 40]. We used this approach for KeepCoool as physicians, practice assistants and those in training differ regarding knowledge and experience. In addition to targeting the professional group and tailoring to baseline knowledge by providing feedback, we used personal address to individualize the learning experience and facilitate behavior change. Such educational approaches have become more widespread with technological advancements [38].

### Strengths and limitations

The content of KeepCoool is based on best-practices and guidelines from several countries. The eLearning addressed all practice personnel. A selection bias cannot be excluded as practices participating in the elearning may differ from those who did not. Contamination between practices was unlikely as they are spread in a larger area and work independently, but cannot be fully excluded. Some practices participated in the eLearning beyond the period defined for the primary outcome. We addressed this in the Euclidian model with 2 million temperature data from up to 4.5 months. For the effectiveness, we chose a strict primary outcome according to recommendations for vaccine storage, yet slightly less strict outcomes might be as appropriate for modern vaccines (e.g., in target range 95% of the time while always above the freeze threshold). Data on changes of refrigerators or practice personnel were not obtained to not burden busy practice personnel. However, we assured that only personnel who had participated in the baseline evaluation were provided access to the elearning. The Euclidian distance used the +5°C median as guidelines recommend to target 5°C when adjusting refrigerators. No long-term monitoring, e.g. after 6 to 12 months, and room temperature monitoring were performed but recommended for future studies.

Overall, the eLearning KeepCoool showed high learning effectiveness [27] and significant improvements regarding the vaccine cold chain. The freely available eLearning allows for the nationwide scaling up to all personnel and practices vaccinating: https://keepcoool.ukbonn.de/.

## Supporting information

S1 Checklist TREND statement checklist. (PDF)

S1 File Includes both the supporting tables and figures of the manuscript. (PDF)
