# Supplementary material for: Effectiveness of the online-eLearning program KeepCoool at improving the vaccine cold chain in general practices
Source: PLoS One. 2024 Apr 16;19(4):e0301847. doi: 10.1371/journal.pone.0301847 (PMC11020848; doi:10.1371/journal.pone.0301847)
Supplement: S1 File — (PDF) [file pone.0301847.s002.pdf]

Effectiveness of the Online-eLearning Program KeepCoool at Improving the Vaccine Cold Chain in General Practices

Supplemental Material

Anika Thielmann[1], Marie-Therese Schmitz[2], Thomas Welchowski[1,2], Birgitta Weltermann[1]*

[1] Institute for Family Medicine and General Practice, University Hospital Bonn, Bonn, Germany

[2] Department of Medical Biometry, Informatics and Epidemiology, Faculty of Medicine, University of Bonn, Bonn, Germany

*Corresponding author

E-Mail: birgitta.weltermann@ukbonn.de

This Supplemental material includes additional information about the regression model described in Article Section "Statistical Analysis". Section 1.1.1. shows the estimated coefficients for the distribution parameter mode. The mode of the regression model represents the most common associations between covariate indicators to temperatures of vaccine refrigerators. Covariates are indicators of for intervention group (X_1), weekend (X_2), participation of physicians (X_3), number of physician assistants (X_4) and joint practice (X_5). The leftmost column in each table describes the specification of the estimated coefficient. Intercept is the average value of the temperature, if all covariates are set to their reference values. Then the covariate main effects are described. Due to large samples size of the data set additional interaction effects with two and three variables could estimated. These are given in Section 1.1.2. for the mode distribution parameter. The descriptions of interaction effects use the abbreviated values X with subscript for the respective covariates. In addition to point estimates in Column "Coefficient" 95 % confidence intervals are given in the next two columns. Then the adjusted p-values for the hypothesis that the effect is zero are displayed. P-values were adjusted by false discovery rate multiple testing correction.

1.1.1. Results of regression model: Distribution parameter mode with identity link

Parameters of variables	Coefficient	Lower 95 % confidence interval	Upper 95 % confidence interval	Adjusted p-value
Intercept	-10,968	-11,044	-10,892	$< 10^{-293}$
Intervention group with reference level “no” (X_1)	12,704	12,626	12,782	$< 10^{-293}$
Weekend with reference level “no” (X_2)	-0,087	-0,098	-0,075	$6.31 * 10^{-52}$
Participation of physician with reference level “no” (X_3)	12,647	12,526	12,768	$< 10^{-293}$
Two participating physician assistants with reference level “1” (X_4)	6,711	6,681	6,741	$< 10^{-293}$
Joint practice with reference level “no” (X_5)	17,908	17,833	17,983	$< 10^{-293}$
Interaction between X_3 and X_4	-5,351	-5,407	-5,296	$< 10^{-293}$
Interaction between total number of participants and X_5	-5,439	-5,467	-5,411	$< 10^{-293}$
Interaction between X_1 and X_2	-0,226	-0,243	-0,209	$8.75 * 10^{-147}$
Interaction between X_1 and X_3	-18,276	-18,399	-18,153	$< 10^{-293}$
Interaction between X_1 and X_4	-5,203	-5,234	-5,171	$< 10^{-293}$
Interaction between X_1 and X_5	-15,433	-15,51	-15,356	$< 10^{-293}$

1.1.2. Results of regression model: Distribution parameter mode with identity link

Parameters of variables	Coefficient	Lower 95 % confidence interval	Upper 95 % confidence interval	Adjusted p-value
Interaction between X_1, X_2 and X_3	0,014	0,007	0,02	$1.94*10^{-5}$
Interaction between X_1, X_2 and X_4	0,038	0,03	0,045	$4.47*10^{-23}$
Interaction between X_1, X_2 and X_5	0,105	0,099	0,112	$1.86*10^{-231}$
Interaction between X_1, X_3 and X_4	8,412	8,355	8,469	$< 10^{-293}$
Interaction between X_1, X_3 and X_5	2,242	2,212	2,271	$< 10^{-293}$
Interaction between X_1, X_4 and X_5	6,998	6,968	7,028	$< 10^{-293}$

1.2.1 Results of regression model: Distribution parameter scale with log link

Section 1.2. gives the analogous coefficient estimates for the distribution parameter scale. This parameter controls how the variance is affected by the covariates. In contrast to Section 1.1. the link between the scale parameter and the coefficients is logarithmic. For easier interpretation the estimates in the second Column includes the exponential transformed coefficients. Given the other covariates remain constant, those values give the multiplicative change, e. g. participation of a physician increased the variance by 25.8 %. Section 1.2.2 continues this table with additional interaction effects.

Parameters of variables	Exponential of coefficient	Lower 95 % confidence interval	Upper 95 % confidence interval	Adjusted p-value
Intercept	25,886	25,111	26,686	$< 10^{-293}$
Intervention group with reference level "no" (X_1)	0,245	0,237	0,253	$< 10^{-293}$
Weekend with reference level "no" (X_2)	0,996	0,989	1,002	0.216
Participation of physician with reference level "no" (X_3)	1,258	1,199	1,32	$1.13*10^{-20}$
Two participating physician assistants with reference level "1" (X_4)	0,49	0,482	0,499	$< 10^{-293}$
Joint practice with reference level "no" (X_5)	0,002	0,002	0,002	$< 10^{-293}$
Interaction between X_3 and X_4	0,752	0,732	0,771	$1.23*10^{-104}$
Interaction between total number of participants and X_5	8,892	8,808	8,978	$< 10^{-293}$
Interaction between X_1 and X_2	0,859	0,847	0,871	$3.4*10^{-99}$
Interaction between X_1 and X_3	3,125	2,975	3,282	$< 10^{-293}$
Interaction between X_1 and X_4	1,901	1,868	1,935	$< 10^{-293}$
Interaction between X_1 and X_5	19,085	18,544	19,643	$< 10^{-293}$

1.2.2 Results of regression model: Distribution parameter scale with log link

Parameters of variables	Exponential of coefficient	Lower 95 % confidence interval	Upper 95 % confidence interval	Adjusted p-value
Interaction between X_1, X_2 and X_3	0,956	0,95	0,962	$5.91*10^{-50}$
Interaction between X_1, X_2 and X_4	1,033	1,026	1,041	$2.46*10^{-19}$
Interaction between X_1, X_2 and X_5	1,106	1,1	1,112	$1.16*10^{-293}$
Interaction between X_1, X_3 and X_4	0,529	0,515	0,543	$< 10^{-293}$
Interaction between X_1, X_3 and X_5	0,452	0,447	0,457	$< 10^{-293}$
Interaction between X_1, X_4 and X_5	0,314	0,31	0,318	$< 10^{-293}$

1.3. Results of regression model: Distribution parameters for skewness

This section describes covariate free estimates of left and right tail skewness of the temperature for refrigerators. Even though that both estimates of skewness are significantly different due to non-overlapping confidence intervals, the magnitude of the differences between left and right tail are small. Thus the estimated distribution is near symmetry. In addition, the tails of the distribution are a bit thicker in comparison to a normal distribution. A normal distribution would have left and right tail values of two.

Parameters of variables	Exponential of coefficient	Lower 95 % confidence interval	Upper 95 % confidence interval	Adjusted p-value
Left tail	1,991	1,988	1,993	$< 10^{-293}$
Right tail	1,839	1,838	1,841	$< 10^{-293}$

1.4. Results of regression model: Estimated curve of duration time

This section shows the estimated P-splines of duration in months on the observed times. The curve in Figure S1 fluctuates around zero with cyclical behaviour. The estimated fluctuations increase after three months compared to the effects before three months. However, magnitude of those effects is still small with maximal absolute changes of 0.4 °C and represents an expected fluctuation.

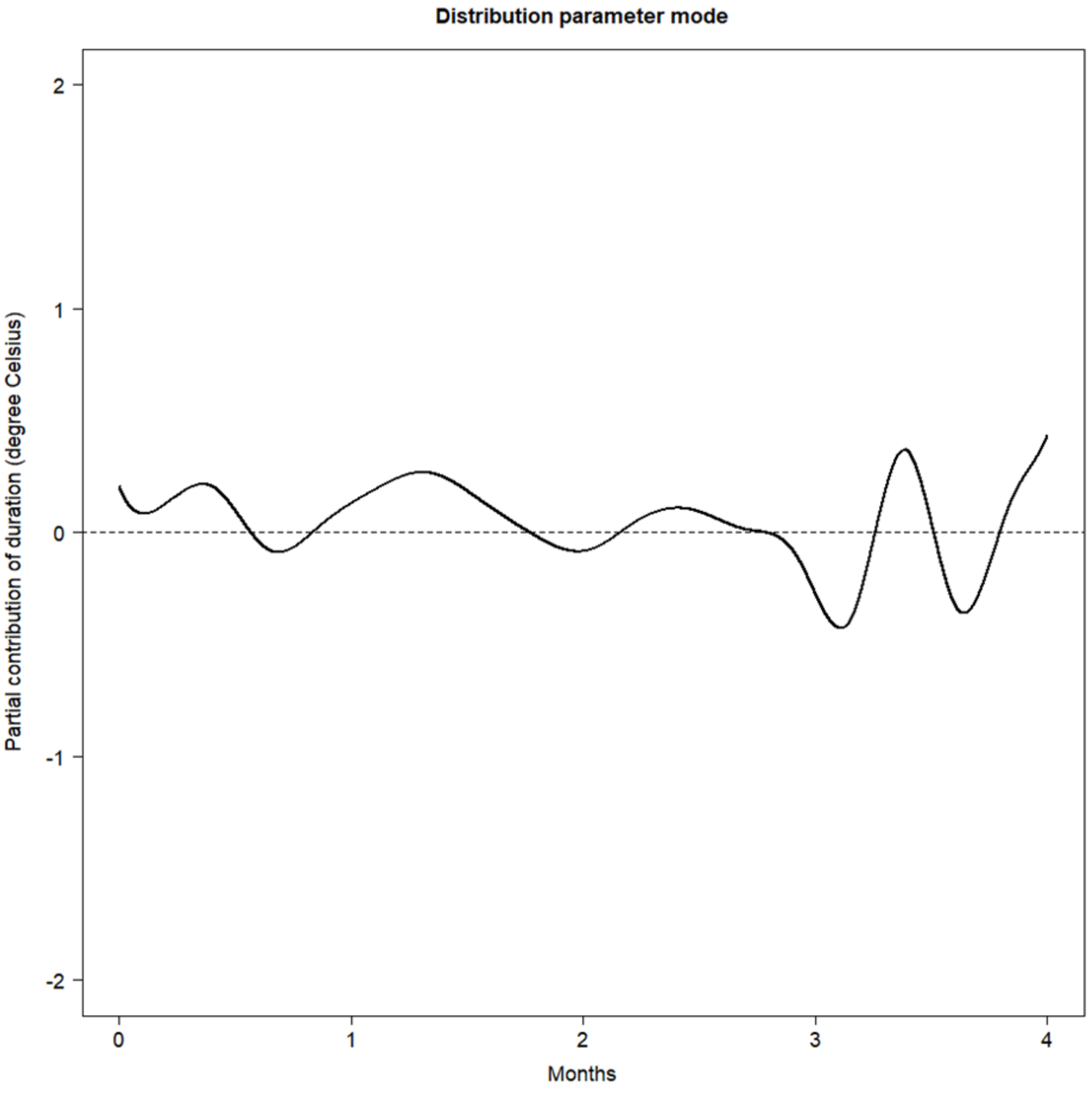

Figure S1: Estimated P-Spline contribution to mode of the temperature of refrigerators (°C)

The following Figure S2 shows the estimated P-Spline effects of duration to the scale of temperature of refrigerators. In the beginning period up to one month of the variability of the temperature was largest. Clearly, the variability of the temperatures decreases for later observations. This is likely due to participants´ learning effects.

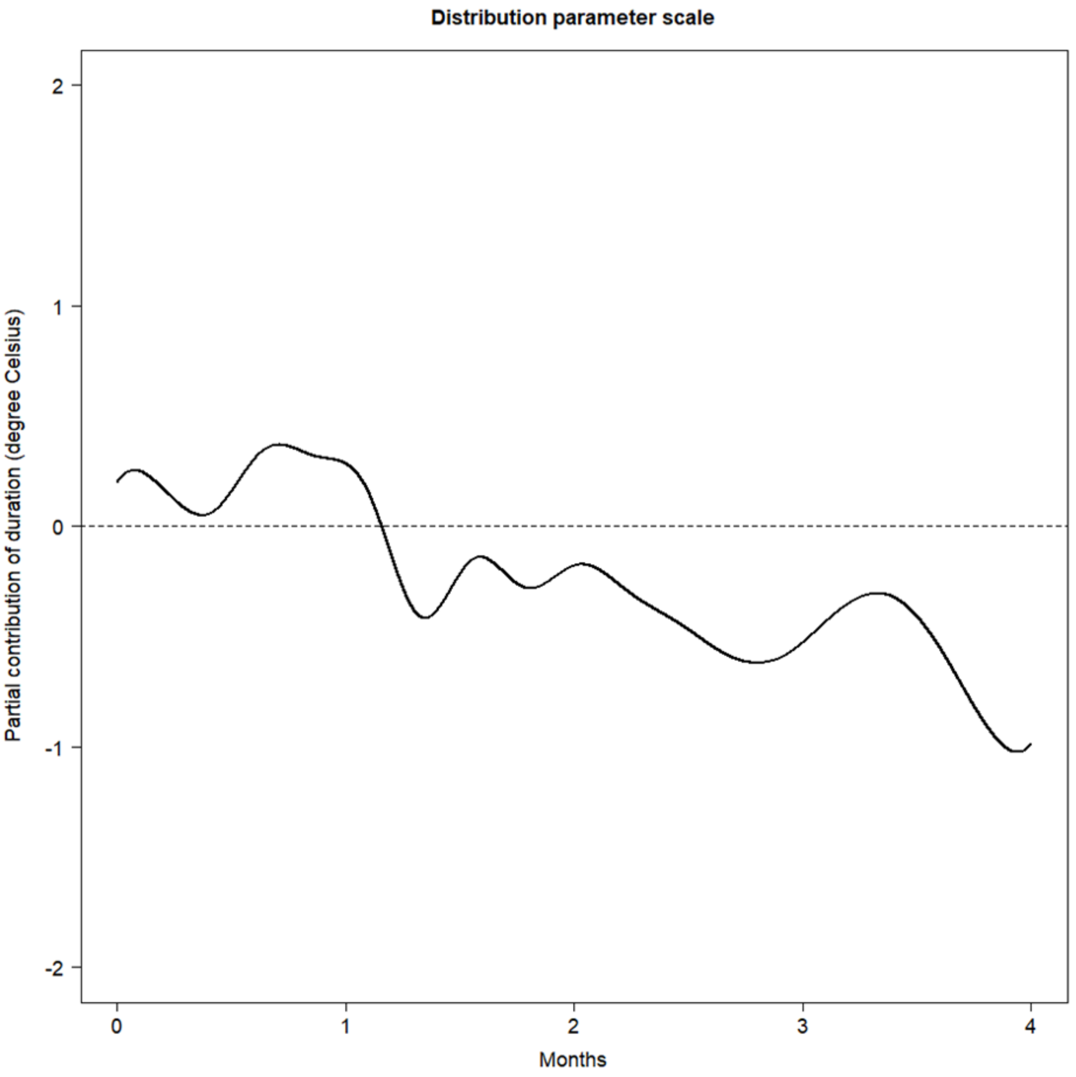

Figure S2: Estimated P-Spline contribution to scale of the temperature of refrigerators (°C)

1.5. Model diagnostics

Quantile residuals were computed from the estimated model on the complete data set to investigate goodness of fit. In case of a well-fitting model the quantile residuals can be shown to be standard normal distributed. Both graphs at the upper half of Figure S3 show that the residuals fluctuate randomly around zero (mean -0.004). The density estimate and the normal

Q-Q plot in the lower part of Figure S3 indicate that the quantile residuals are similarly distributed as a standard normal distribution (variance 0.999). The density estimate in the lower left part is nearly symmetric (coefficient of skewness 0.019). Kurtosis is higher than for a standard normal distribution (excess kurtosis 0.755). The lower right Normal Q-Q plot shows that the estimated quantiles of the quantile residuals match very well those of the standard normal distribution in the range between -3 and 3. Quantile residuals outside this range do not fit. Overall, the fit of the model to the data is very good as indicated by the Filliben correlation coefficient of 0.996.

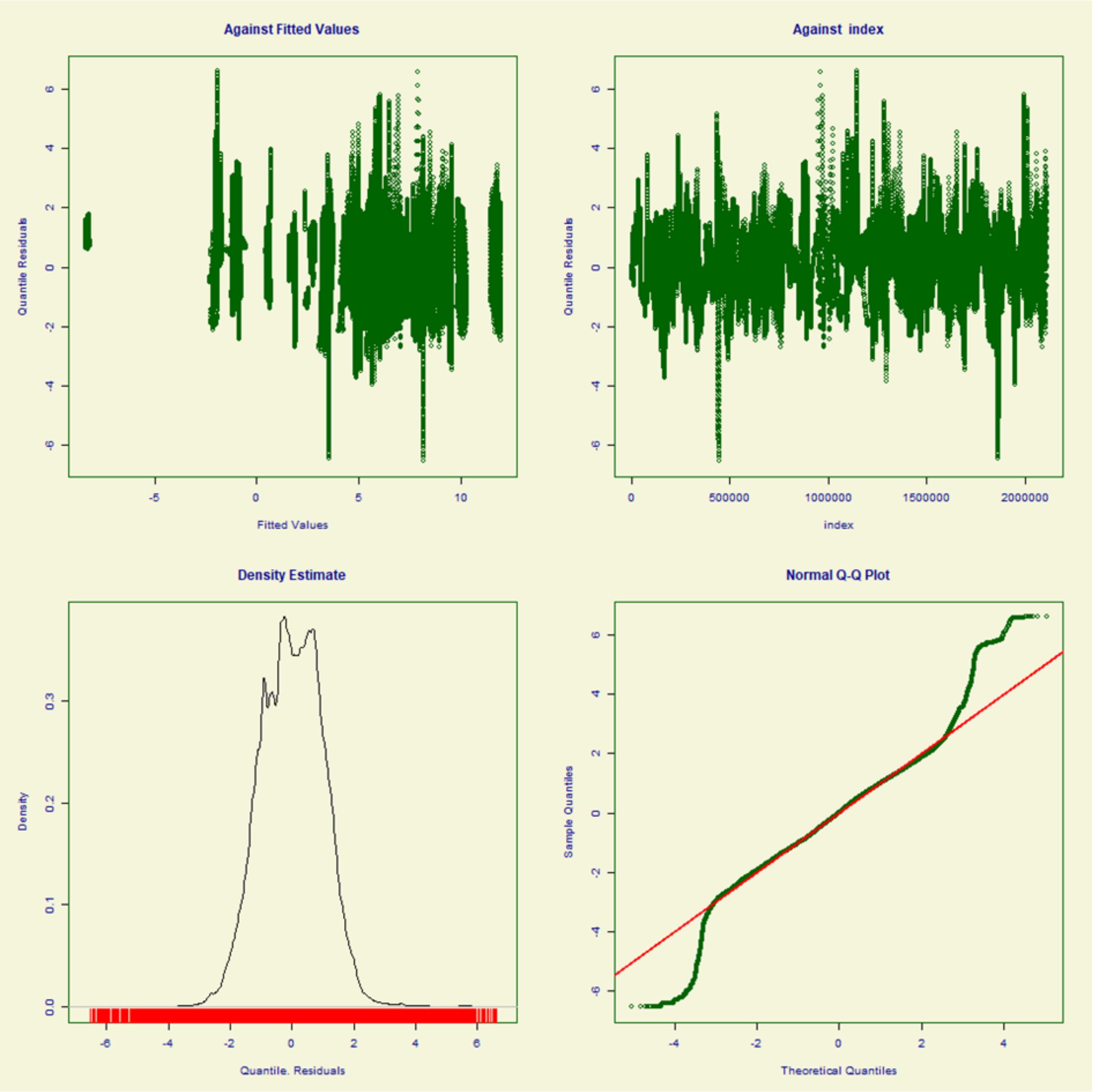

Figure S3: Model diagnostics of the estimated generalized additive model for location, scale, and shape
